# Phospholipids reduce gastric cancer cell adhesion to extracellular matrix *in vitro*

**DOI:** 10.1186/1471-230X-4-33

**Published:** 2004-12-29

**Authors:** Marc Jansen, Karl-Heinz Treutner, Britta Schmitz, Jens Otto, Petra Lynen Jansen, S Neuss, Volker Schumpelick

**Affiliations:** 1 Department of Surgery, University Clinic, Pauwelsstr. 30, 52057 Aachen, Germany; 2 Interdisciplinary Centre of Clinical Research (IZKF) Biomat; University Clinic, Pauwelsstr. 30, 52057 Aachen, Germany; 3 Institute of Pathology, University Clinic, Pauwelsstr. 30, 52057 Aachen, Germany

## Abstract

**Background:**

Nidation of floating tumour cells initiates peritoneal carcinosis and limits prognosis of gastro-intestinal tumours. Adhesion of tumour cells to extracellular matrix components is a pivotal step in developing peritoneal dissemination of intraabdominal malignancies. Since phospholipids efficaciously prevented peritoneal adhesion formation in numerous animal studies we investigated their capacity to reduce adhesions of gastric cancer cells to extracellular matrix components (ECM).

**Methods:**

Human gastric cancer cells (NUGC-4, Japanese Cancer Research Resources Bank, Tokyo, Japan) were used in this study. Microtiter plates were coated with collagen IV (coll), laminin (ln) and fibronectin (fn). Non-specific protein binding of the coated wells was blocked by adding 1% (w/v) BSA (4°C, 12 h) and rinsing the wells with Hepes buffer. 50.000 tumour cells in 100 μl medium were seeded into each well. Beside the controls, phospholipids were added in concentrations of 0.05, 0.1, 0.5, 0.75 and 1.0/100 μl medium. After an incubation interval of 30 min, attached cells were fixed and stained with 0.1% (w/v) crystal violet. The dye was resuspended with 50 μl of 0.2% (v/v) Triton X-100 per well and colour yields were then measured by an ELISA reader at 590 nm. Optical density (OD) showed a linear relationship to the amount of cells and was corrected for dying of BSA/polystyrene without cells.

**Results:**

The attachment of gastric cancer cells to collagen IV, laminin, and fibronectin could be significantly reduced up to 53% by phospholipid concentrations of 0.5 mg/100 μl and higher.

**Conclusion:**

These results, within the scope of additional experimental studies on mice and rats which showed a significant reduction of peritoneal carcinosis, demonstrated the capacity of phospholipids in controlling abdominal nidation of tumour cells to ECM components. Lipid emulsions may be a beneficial adjunct in surgery of gastrointestinal malignancies.

## Background

In the treatment of gastro-intestinal cancer the detection of free, isolated tumour cells in the peritoneal cavity serve as a prognostic marker for postoperative survival [1-4]. Since surgery frequently proofs insufficient for tumour control, numerous additional treatments have been evaluated. A pivotal step in developing peritoneal dissemination seems to be adhesion of tumour cells to mesothelial cells or extracellular matrix components [5-7]. Experimental studies suggest that peritoneal metastases tend to occur in areas of injured peritoneum [8]. Cell-matrix interactions are promoted by transmembrane receptors with integrins as a major family. Many attempts were made to inhibit tumour cell attachment by antibodies against adhesion molecules [9], dextran sulphate [10], or sodium hyaluronate [11] with different results concerning tumour adhesion.

Phospholipids, polar phosphoric acid di-esters, are natural constituents of the abdominal fluid. The substance is able to form a lubricant layer on the peritoneal surface [12]. Additionally, integrin function, particularly in control of cell motility is affected by exogenous addition of phospholipids (e.g. gangliosides) [13,14]. Intraperitoneal use of phospholipids (PL) led to a significant decrease of adhesion formation especially at sites of peritoneal injury [15,16]. The objective of the underlying *in vitro *study was focused on the influence of phospholipids on adhesion of gastric cancer cells to extracellular matrix components with broad reactivity to several integrins. Collagen IV (coll IV), and laminin (ln) are main components of the basement membrane and fibronectin (fn) plays an important role in wound healing [17,18].

## Methods

### Tumour cells

The human gastric cancer cell line NUGC-4 was purchased from the Japanese Cancer Research Resources Bank (Tokyo, Japan). The cells were maintained in monolayers in tissue culture flasks (75 cm^2^, Falcon, Becton Dickinson-Gambil, Heidelberg, Germany) in RPMI 1640 medium (GIBCO, Karlsruhe, Germany), supplemented with 10% foetal bovine serum (GIBCO), penicillin and streptomycin (GIBCO). Cell cultures were incubated at 37°C in a humidified atmosphere of 5% CO_2 _in air. Cells were passaged after treatment with 0.125% trypsin for 6 min. The cells were pelleted after centrifugation for 10 min at 200 g, suspended in 20 ml PBS, and pelleted. The cell pellet was resuspended in 30 ml complete medium and seeded with a splitting ratio of 1:3. Only cells from three passages were used for the experiments.

### Extracellular matrix (ECM) components

Flat-bottom polystyrene microtiter plates (Becton Dickinson, Heidelberg, Germany) were coated for adhesion experiments. The purified ECM components were dissolved in PBS with the following concentrations: coll IV – 2,5 μg/ml (Biomol, Hamburg, Germany), fn – 10 μg/ml (Boehringer, Mannheim, Germany), ln – 50 μg/ml (Boehringer, Mannheim, Germany). We found these concentrations to be optimal in foregoing dilution series. They were added to the wells and incubated at 4°C for 24 hours (coll IV, fn), or at 37°C for 45 min in a humidified atmosphere of 5% CO_2 _in air (ln), respectively. Nonspecific protein binding of the coated wells was blocked by adding 1% (w/v) BSA (4°C, 12 h) and rinsing the wells with Hepes buffer.

### Adhesion assay

For adhesion experiments gastric cancer cells were detached with collagenase I (15 min, 37°C, Worthington, Freehold, USA), washed once with RPMI 1640, centrifuged (200 g for 10 min), resuspended in RPMI 1640, and preincubated for 30 min in a humidified atmosphere of 5% CO2 in air (37°C). Fifty thousand tumour cells in 100 μl medium were seeded into each well. Evaluation of adherent cells was performed using crystal violet staining according to the method described by Aumeilley et al., and Tietze et al. [19,20]. After an incubation period of 30 min the supernatant with non-adherent cells was removed by two washes with warmed RPMI 1640. Attached cells were fixed with 30% (v/v) methanol/ethanol for 15 min at room temperature. Cells were stained with 0.1% (w/v) crystal violet (Sigma, Hamburg, Germany), extensively washed with distilled water, and dried at room temperature. The dye was resuspended with 50 μl of 0.2% (v/v) Triton X-100/well and colour yields were then measured using an ELISA reader at 590 nm (Titertek Multiscan Plus MKII, Flow Laboratories GmbH, Meckenheim, Germany). Optical density (OD) showed a linear relationship to the amount of cells between 1 × 10^3 ^and 5 × 10^4 ^cells per well, as determined by a dilution series.

Control dying of BSA/polystyrene without cells led to Optical Density (OD) values of 0.01–0.07. These values were subtracted from those obtained in the experiments.

### Phospholipids

After complete preparation of the tumour cell suspension, the PL solution was added in the following concentrations: 0.05, 0.1, 0.5, 0.75, and 1 mg per 100 μl medium. The concentrations used were correlated to our in vivo experiments. The phospholipid solution consists of phosphatidylcholine 70% by weight, phosphatidylethanolamine 15% by weight, neutral lipids 8% by weight, sphingomyelin <3% by weight and lysophosphatidylcholine <3% by weight.

### Statistical analysis

All experiments were performed three times in quadruplicate. The data are expressed as means +/- standard error of the mean (SEM). Student's t-test for unpaired data was used for statistical analysis. Differences were regarded as significant for p values < 0.05.

## Results

The analysis of tumour cell adhesion to BSA 1% resulted in a mean extinction of 0.27 (SEM 0.01) at 590 nm. Coating with ln and fn led to a nearly twofold increase of tumour cell adhesion with mean values of 0.59 (0.03, ln) and 0.63 (0.03, fn). The cancer cells showed a most pronounced adhesion to coll IV with a mean extinction of 0.97 (0.02).

The tumour cell adhesion to ln registered after addition of PL was significantly reduced. The effect was concentration dependent compared to the controls. Even the minimum amount of PL 0.05 mg/100 μl led to a reduced extinction of 0.4 (0.01). Treatment with 0.1 or 0.5 mg/100 μl PL revealed extinction values of 0.32 (0.02) and 0.28 (0.02), respectively. The maximum effect could be demonstrated with 0.75 mg/100 μl PL with an extinction of 0.24 (0.02). The relative reduction of tumour cell adhesion compared to the control amounts to 59%. Treatment with 1 mg/100 μl PL showed no further decrease of tumour cell adhesion to ln. The mean extinction was 0.26 (0.01) (table 1).

**Table 1:** Influence of different phospholipid concentrations on adhesion of gastric cancer cells to laminin. Optical density (OD) measured in an ELISA reader at 590 nm

	Extinction at 590 nm	SEM	p
Control	0.59	0.03	
PL 0.05 mg/well	0.4	0.01	p < 0.05
PL 0.1 mg/well	0.32	0.02	p < 0.05
PL 0.5 mg/well	0.28	0.02	p < 0.05
PL 0.75 mg/well	0.24	0.02	p < 0.05
PL 1 mg/well	0.26	0.01	p < 0.05

The tumour cell adhesion on fn could not be reduced significantly with low concentrations of Pl. Addition of 0.05 mg/100 μl PL and 0.1 mg/100 μl resulted in a slight reduction of the extinction with mean values of 0.59 (0.02) and 0.59 (0.01). However, a significant reduction of tumour cell adhesion could be observed after treatment with 0.5 mg/100 μl PL, 0.42 (0.02); as well as with 0.75 mg/100 μl PL (0.39 (0.02)) and 1 mg/100 μl PL (0.38 (0.02)). We found a similar situation compared to ln with equal effects of 0.75 mg/100 μl and 1 mg/100 μl PL indicating that the maximum influence on adhesion is reached. The relative reduction of tumour cell adhesion compared to the control values amounts to 40% (table 2).

**Table 2:** Influence of different phospholipid concentrations on adhesion of gastric cancer cells to fibronectin. Optical density (OD) measured in an ELISA reader at 590 nm

	Extinction at 590 nm	SEM	p
Control	0.63	0.03	
PL 0.05 mg/well	0.59	0.02	n. s.
PL 0.1 mg/well	0.59	0.01	n. s.
PL 0.5 mg/well	0.42	0.02	p < 0.05
PL 0.75 mg/well	0.39	0.02	p < 0.05
PL 1 mg/well	0.38	0.02	p < 0.05

NUGC-4 gastric cancer cells prominently adhere to collagen IV compared to all other examined extracellular matrix components. The influence of PL on cell adhesion to coll IV was also concentration dependent. The reduction ranged from an extinction of 0.89 (0.01) after administration of 0.05 mg/100 μl PL to a maximum effect after treatment with 1 mg/100 μl PL with a value of 0.44 (0.02) (table 3). In comparison to the control value, this means a reduction of adherent tumour cells of 55%.

**Table 3:** Influence of different phospholipid concentrations on adhesion of gastric cancer cells to collagen IV. Optical density (OD) measured in an ELISA reader at 590 nm

	Extinction at 590 nm	SEM	p
Control	0.97	0.02	
PL 0.05 mg/well	0.9	0.01	p < 0.05
PL 0.1 mg/well	0.74	0.02	p < 0.05
PL 0.5 mg/well	0.61	0.02	p < 0.05
PL 0.75 mg/well	0.45	0.03	p < 0.05
PL 1 mg/well	0.44	0.02	p < 0.05

## Discussion

Cell adhesion to the extracellular matrix plays a fundamental role in peritoneal carcinosis. The adhesion is mediated by transmembrane Integrins. Several proteins including fibronectin in the interstitial matrix, laminin and collagen IV in the basement membrane were identified as important ligands [21,22]. Many attempts were made to inhibit tumour cell adhesion by integrin antibodies or competitive inhibitors against specific peptide sequences [23-26]. Gui et al. showed that adhesion of different breast cancer cells to extracellular matrix components could be reduced by specific integrin antibodies [27]. However, different antibodies for different cell lines were necessary according to the expression of specific integrins on the cell surface. Haier et al. could find different adhesive capacities to collagen I in two subtypes of the HT-29 colon carcinoma cell line. The cells with a very limited capability to induce hepatic metastases showed a significant higher rate of adhesions compared to those inheriting a high potential for involvement of the liver [28]. The influence of three examined phosphotyrosine kinase inhibitors on integrin mediated tumour cell adhesion to collagen I was unspecific. Dennis et al. found different cell-surface receptors responsible for cell attachment to fibronectin and collagen as compared to laminin. They concluded with the hypothesis that specific glycolipids may be receptors for interaction with fibronectin [29].

In our experiments the reduced rate of cell attachment in the presence of phospholipids was independent from the extracellular matrix. A similar effect on intraperitoneal tumour growth was described by Jacobi et al. who could demonstrate that taurolidine/heparin and povidone iodine lead to a significant reduction of tumour cell growth *in vitro *as well as a reduction of tumour weight after intraperitoneal tumour injection [30]. Predominantly the result seems to be a attributed to the cytotoxic effect of the used substances and benefits to a lesser degree from adhesion prevention. Other substances used to prevent adhesion failed in the treatment of inhibiting tumour cell attachment. Sodium hyalunorate increased the metastatic potential of colo-rectal tumour cells, probably mediated by the CD44 receptor [31]. Dextran sulphate resulted in reduced tumour cell nidation at sites of injury to abdominal wall in mice [10,32]. However, several side effects were described in the use of dextrane for adhesion prevention. Main problems were oedema, pleura effusion, life-threatening coagulation disorders and severe allergic reactions [33,34]. Phospholipids, polar phosphoric acid di-esters, are natural constituents of the abdominal cavity fluid and cell membranes.

The hypothesis is that phospholipids form a lubricant layer on the peritoneum by binding with its negatively charged cholin branch chain to the positively charged peritoneal surface [12,14,35]. Phospholipids cover the entire peritoneal membrane by a thin fluid layer. By separating tumour cells from the peritoneal surface they proved to significantly reduce peritoneal carcinosis. Phospholipipds seem to reduce the expression of integrins and adhesion molecules on the cell surface to the effect that adhesions can be prevented reducing tumour cell attachment independent from their origin [15,16].

The in situ tumour cell – ECM interaction is influenced by adhesive and non-adhesive ECM components and can be understood as a three dimensional network [36]. Therefore the *in vitro *experiments with tumour cells as soluble agents added to ECM immobilized onto plastic surfaces cannot appropriately mimic the situation in situ. Recently we found that phospholipids significantly reduce the attachment area and the tumour volume of peritoneal carcinosis caused by the colonic cancer cell line DHD/K12/TRb in rats. These results were supported by a prolonged survival rate of the treated animals as compared to the control group. Additionally, we found a similar effect of phospholipids on the adhesion of the human rectal cancer cell line HRT-18 on the same ECM-components *in vitro *[37]. Consistent with results of other groups, the tumour cell attachment was found predominantly in areas of previously injured peritoneum [5,6,38,39].

We performed this study to ascertain the results of the foregoing animal experiments and to demonstrate the influence of phospholipids to three different ECM components, even though matrices of collagen IV, laminin and fibronectin alone may not be predictive of peritoneal membrane nidation.

## Conclusion

These results, within the scope of additional experimental studies on mice and rats which showed a significant reduction of peritoneal carcinosis, demonstrated the capacity of phospholipids in controlling abdominal nidation of tumor cells to ECM components. Lipid emulsions may be a beneficial adjunct in surgery of gastrointestinal malignancies.

## Competing interests

The work was financially supported by Fresenius Kabi, Bad Homburg, Germany.

The results are part of an international patent application.

## Pre-publication history

The pre-publication history for this paper can be accessed here:


